# Tooth-implant connection with fixed partial dentures in partially edentulous arches. A retrospective cohort study over an 11.8 year observation period

**DOI:** 10.4317/jced.58170

**Published:** 2021-07-01

**Authors:** Gregor-Georg Zafiropoulos, Moosa Abuzayeda, Adel A. Al-Asfour, Syed-Saad-Bin Qasim, George Pelekos, Colin-Alexander Murray

**Affiliations:** 1 Professor, Kuwait University, Faculty of Dentistry, Department of Surgical Sciences, Kuwait; 2 Assoc. Professor, MBR University, College of Dentistry, Department of Prosthodontics, Dubai, UAE; 3 Assoc. Professor, Kuwait University, Faculty of Dentistry, Department of Surgical Sciences, Kuwait; 4 Ass. Professor, Kuwait University, Faculty of Dentistry, Department of Bioclinical Sciences, Kuwait; 5 Ass. Professor, Hong Kong University, Faculty of Dentistry, Division of Periodontology, Hong Kong; 6 Professor, University of Sharjah, Department of Preventive and Restorative Dentistry, UAE

## Abstract

**Background:**

The fixed tooth-implant connection remains a controversial issue. This private practice-based retrospective study aimed to evaluate the clinical outcomes of a contemporary fixed partial denture (FPD) design for connecting natural teeth and implants (TI-FPD), over an 11.8 years observation period.

**Material and Methods:**

The data of 91 partially edentulous patients (44 males and 47 females, mean age of 47.7 years) treated with a newly designed TI-FPD retained on 1 implant and 1 natural tooth were analyzed retrospectively. Teeth were covered with electroformed copings and a CAD/CAM made bridge was fixed over the abutments with provisional cement. Two different implant systems were used: Camlog (N=22; anterior areas) and Straumann tissue level (N=69; posterior areas).

**Results:**

The survival rate for both implants and teeth was 100%. 19/21 (90%, 95%CI 82–95%), 16/21 (66%, 95%CI 66–84%), and 16/21 (66%, 95%CI 66–84%) patients were free of biological complications after 5 years, 10 years, and 15 years post-loading, respectively. 23/35 (90%, 95%CI 54–74%), 21/35 (61%, 95%CI 50–70%), and 21/35 (61%, 95%CI 50–70%) were free of technical complications following 5 years, 10 years, and 15 years post loading, respectively.

**Conclusions:**

Despite limitations of the study, the findings demonstrated that the use of a recently designed TI-FPD could be used for the tooth-implant connection in cases of partial edentulism and this may widen the treatment modalities by reducing the cost and need for extensive bone tissue augmentations. Further controlled longitudinal studies with larger patient groups are needed.

** Key words:**Tooth–implant connection, dental implant, fixed partial dentures, complications, implant prosthodontics.

## Introduction

Implant restorations in partially edentulous patients are a predictable treatment modality for missing dentition with respect to functionality and aesthetics. In cases of partial edentulism in which placement of an adequate number of supporting implants is either not possible without augmentative surgery, patient´s unwillingness for augmentive surgery or because of economic reasons, tooth-to-implant retained fixed partial dentures (TI-FPD) could be a possible alternative treatment. However, the combined connection of teeth and implants remains a controversial issue and subject to discussion (1-7). Due to the absence of clear guidelines and conflicting reports, the clinician is faced with the controversy of whether connecting implants to natural teeth is a valid treatment option with an adequate success and/ or survival rate (1,8).

A frequent argument is that the difference in mobility between the tooth and the implant increases the risk for clinical failures and complications such as fracture of mechanical parts, a higher incidence of caries at the crown margin or tooth intrusion (1,2). The difference in movement of a tooth in good periodontal health and an osseointegrated implant can be five to twenty times greater. This has resulted in substantial debate as to whether teeth should be extracted for the sake of avoiding tooth-implant connection (9-11). The risk of intrusion of natural teeth, when combined with implants to support a fixed partial denture (FPD), means that a decision to extract is frequently taken in order to avoid tooth-implant connections. The aim of this study was to evaluate the clinical outcomes of a novel TI-FPD design over a 16-year observation period to determine if it could be an option in routine clinical practice.

## Material and Methods

-Study design and population

In this private practice-based, non-randomized study the data of 91 prospectively recruited, partially edentulous patients (44 males and 47 females, mean age = 47.7 years) treated with a newly designed TI-FPD retained on 1 implant and 1 natural tooth during 2000 to 2016 were analyzed retrospectively (Table 1). The patients treated in this way requested treatment with a fixed restoration. However, they did not wish augmentative surgical procedures including sinus lift or due to financial limitations the treatment would not be possible with an FPD retained on 2 or 3 implants.

**Table 1:**
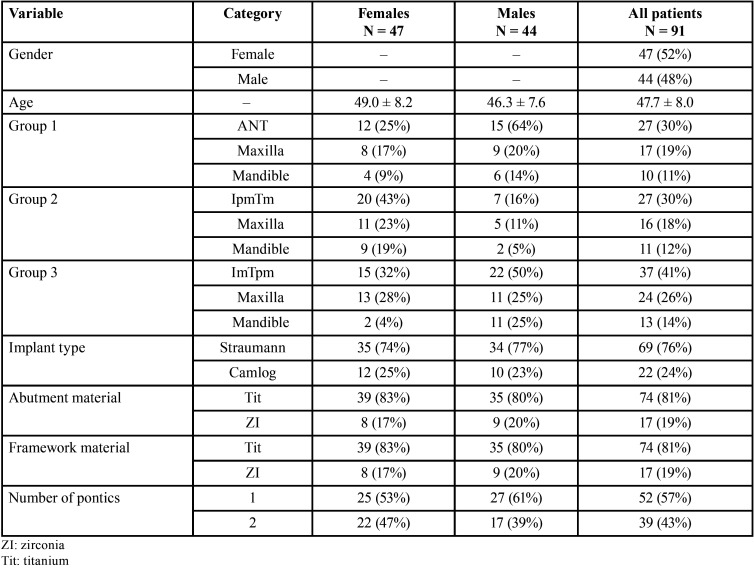
Patient demographics and implant details [mean ± standard deviation, number (N), percentage (%)].

The inclusion criteria were the treatment of 1) unilateral partially edentulous area with 1 or 2 neighboring missing teeth; 2) the willingness and ability to provide informed consent; 3) patients demonstrating good oral hygiene and compliance [mean number of surfaces with plaque (PL) less than 10% and mean number of periodontal pockets with bleeding on probing (BOP) less than 8%]; 4) teeth adjacent to the implant area were free of overhangs or deficient restoration margins or caries; 5) patients had to be non-smokers, or had stopped using all tobacco products for at least 1 year before undergoing treatment; 6) The TI-FPD had antagonist occlusion.

The exclusion criteria were 1) pregnancy; 2) uncontrolled diabetes; 3) use of an anticoagulant drug; 4) history of aggressive periodontitis; 5) bruxism and/or tooth clenching; 6) immediate implant placement; 7) 3rd molar areas; 8) in cases with bilateral partial edentulism treated according to the protocol reported hereby, only one of the edentulous areas was randomly selected and included in this study; 9) no immediate implant placement and loading.

In compliance with the Declaration of Helsinki (1996, i.e. 2000), patients were informed orally and in writing, i.e. informed consent, about the planed treatment procedures and given at least 2 weeks for consideration. Patients had the right to withdraw consent and to interrupt treatment at any time without reprisal. For each one patient, the treatment plan and the informed consent were approved by the national health authorities (KZV -Association of Statuary Health Insurance Dentists, Germany), which also approved the analysis of the cases and the publication of the results (Dental Council North-Rhine; Germany; No: RA 232.20 AK-cls). All periodontal, implant surgical, and prosthetic treatment procedures were performed by one of the authors (GGZ).

-Groups

Restoration sites included both arches. For our implant-location analyses (Table 2), anterior restorations in either arch was considered together as a single group. The restorations were grouped according to the area of restoration (anterior, premolar, or molar), implant site and tooth location as follows:

**Table 2:**
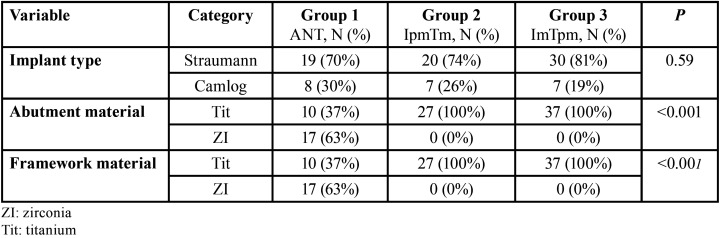
Group comparisons.

Group 1 (ANT): anterior TI-FPD. Maxillary anterior restorations had customized zirconia implant abutments with zirconia frameworks, whereas mandibular anterior restorations had prefabricated titanium (Tit) implant abutments with chromium-cobalt (CrCo) frameworks.

Group 2 (IpmTm): posterior TI-FPD with an implant placed in a premolar site and a molar tooth.

Group 3 (ImTpm): posterior TI-FPD with an implant placed in a molar site and a premolar tooth, (Table 3).

**Table 3:**
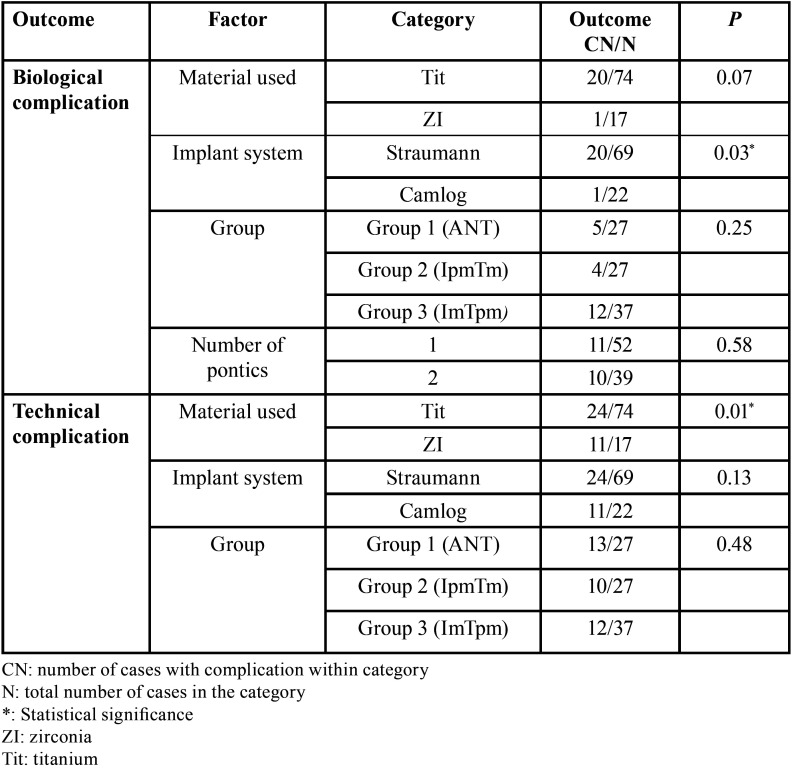
Factors associated with restoration survival outcomes.

-Treatment history

All patients had a history of periodontitis with an attachment loss (AL) of 3-4 mm and had received periodontal treatment (scaling/root planning, flap surgery) and necessary tooth extractions 6 to 7 months before undergoing implant surgery.

In Group 1, Camlog implants (Camlog Biotechnologies GmbH; Basel, Switzerland) were placed and in the posterior areas (Groups 2 and 3) Straumann tissue level implants (Straumann, Basel, Switzerland) were placed. Implant placement was performed with use of implant planning software (Sicat Implant; Sicat, Bonn, Germany) and surgical guides (Surgical Guides; Sicat, Bonn, Germany). Local anesthesia was achieved with 4% articaine HCl injection containing 1:100,000 epinephrine (Ultracain D-S forte; Sanofi-Aventis, Frankfurt/M, Germany). Implant sites were prepared at 875 RPM and implants inserted with a hand-ratchet. Four months after placement, the implants were uncovered.

-Prosthetic treatment

Following 4 months of osseointegration, implants were loaded. After implant uncovering, natural tooth abutments were prepared with a shoulder and a bevel 0.6~1.0 mm sub-gingivally. Natural tooth abutment impressions were recorded with a polyether material (Impregum, 3M ESPE, St. Paul, MI, USA) and a cast fabricated. Electroformed 0.25-mm-thick Auro Galvano Crowns (AGCs) which are gold copings made from Galvano gold with a gold content of greater than 99.9%, (Wieland Dental, Pforzheim, Germany) were fabricated as previously described and cemented onto the tooth abutments with zinc phosphate cement (Harvard Dental, Hoppegarten, Germany).12 Within 2 days, a new impression for the restored tooth abutment and implant was taken with polyether material (Impregum, 3M ESPE, St. Paul, MI, USA) using a system specific implant impression coping. A new master cast was made, a system-specific titanium (Tit) prefabricated implant abutment was selected, and the metal framework milled from a CrCo alloy for posterior or mandibular anterior abutments (Zenotec NP, Wieland, Pforzheim, Germany). In the anterior maxilla, zirconia (ZI) customized implant abutments with a ZI framework were fabricated. After the ZI framework was created, it was milled with ZENO Discs (Wieland Dental, Pforzheim, Germany) and sintered. Fifty-two (57%) of the TI-FPDs had only one pontic and 39 (43%) had two pontics with a modified ridge lap design.

At the try-in session, the framework (CrCo or ZI) was placed over the tooth/implant abutments, and occlusion checked with occlusal records. Subsequently, frameworks were veneered with porcelain (Vintage MP; Shofu, Ratingen, Germany). Implant abutments were mounted on the implants with a transfer key (torque, 35 Nm). Abutment screw openings were filled with a single-component light-cured resin (Fermit; Ivoclar Vivadent, Schaan, Liechtenstein), the patient’s occlusion checked and the TI-FPD finished, polished and cemented with provisional luting cement (TempBond; Kerr, Orange, CA; USA) .

-Maintenance

Following implant placement, patients were prescribed a non-steroidal anti-inflammatory drug for pain relief (diclofenac, 100 mg once a day for 4 days; Novartis, Nuernberg, Germany) and a systemic antibiotic (clyndamycin 600 mg, once a day, 6 days, Ratiopharm, Ulm, Germany). Patients were instructed to rinse twice daily with 0.1% chlorhexidine digluconate solution (Chlorhexamed Fluid, GlaxoSmithKline, Buehl, Germany) starting 1 day before surgery until 1 week after. Sutures were removed after 8 days. Patients were enrolled in supportive periodontal care (SPC) consisting of 4 quarterly follow-up appointments per year. Tooth/implant restorations were polished and oral hygiene instructions were assessed and reinforced. Annually, attachment loss using the crown margins as reference points was measured (data not shown). Every 2 years, TI-FPDs were removed, the abutments cleaned using polishing paste and subsequently the TI-FPDs were re-cemented.

-Complications

At follow-up appointments, patients were analyzed for development of technical and/or biological complications. TI-FPD de-cementation was considered a technical complication except at appointments scheduled for TI-FPD removal.

-Data Analysis

The aims of the analyses were to summarize dataset characteristics, examine associations between variables and examine patient outcomes. The first analyses considered patients’ demographic characteristics and whether implant characteristics varied between patient groups. All variables of interest were categorical in nature, and thus the chi-square test was used for inter-group comparisons. With respect to patient outcomes, biological complications and technical complications were considered. Total follow-up time varied among the patients therefore, survival analyses of these outcomes were undertaken. Time from implant placement to each outcome of interest was calculated. Patients not experiencing an outcome were excluded from the last follow-up time point in analysis of that outcome. Kaplan-Meier analysis were used to calculate the proportion of patients without an outcome occurring throughout the follow-up period. Percentages of patients not yet experiencing each outcome are reported with 95% confidence intervals (CIs). Log rank tests were used to compare time to the outcome between patient groups. Assumptions of the tests were validated and met in all instances. For the Chi-square test, the expected numbers were examined and for the survival analyses, assumptions of proportional hazards were investigated. Statistical analyses were conducted in Stata software (v. 15.1; StataCorp LLC, College Station, TX, USA).

## Results

In the present private practice-based cohort study, 91 partially edentulous areas treated with FPDs fixed with provisional cement using one tooth and one implant as abutments were followed-up [females 47 (51.65%), males 44 (48.35%)]. Fifty-seven (63.64%) of the TI-FPDs were placed in the maxilla and 34 (36.36%) in the mandible. Demographic and implant characteristics for the whole study cohort and each gender group are summarized in Table 1. Categorical variable data are presented as numbers and percentages of patients in each category, whilst continuous variables are presented as means with standard deviations. Males and females were similarly represented in the cohort with a mean age of almost 48 years. During observation time, patients demonstrated good compliance and oral hygiene (mean number of surfaces with plaque (PL) 15% and mean number of pockets with bleeding on probing (BOP) was 9%). Half of patients received a molar implant in association with a premolar tooth abutment. Three-quarters of the implants were Straumann and in >80% of the cases, the abutment and framework were made of Titanium.

The mean post-loading observation time was 11.8 years (range, 4.7–15.2 years). During the observation period, there were no framework or abutment fractures, implant failures, loss of tooth abutments, tooth intrusions, caries or AGC de-cementation events. The patients had a mean of 36 ± 8 (range, 15–46) follow-up appointments (appointment at which the TI-FPD was placed was not included).

Implant and natural tooth location analysis results are reported in Table 2. For the purposes of analysis, the two anterior groups (maxilla and mandible) were combined. No significant differences were found among the 3 groups with respect to implant type. Abutment and framework material did vary among the groups owing to Titanium being used for all patients in the IpmTm and ImTpm groups, whereas only a third of patients in the anterior group, those with mandibular restorations, had titanium abutments with a titanium framework.

The results of complication outcome analyses are reported in Table 4. Almost a quarter of the patients experienced at least one biological complication, whilst more than a third had at least one technical complication. Some patients had more than one type of complication. For natural teeth abutments, periodontal disease with a pocket depth of ≤5 mm was the most common biological complication and TI-FPD de-cementation was the most common technical complication. Of 7 patients presenting with 2 biological complications, 6 had both a natural abutment with recurrent periodontal disease and pocket depth ≤5 mm (AL ≤3 mm) and an implant with mucositis. The remaining patient had an implant with peri-implantitis and a natural tooth abutment with recurrent periodontal disease with a pocket depth >5 mm (AL >3 mm). Two patients had more than one technical complication, both presenting with fracture of the veneering material and TI-FPD de-cementation. With respect to the timing of complications, the 7 patients who had two biological complications had complications detected at similar post-loading time points. For the 2 patients with two technical complications, 1 patient had these at year 1 and year 3, and the other patient had them both at year 5.

**Table 4:**
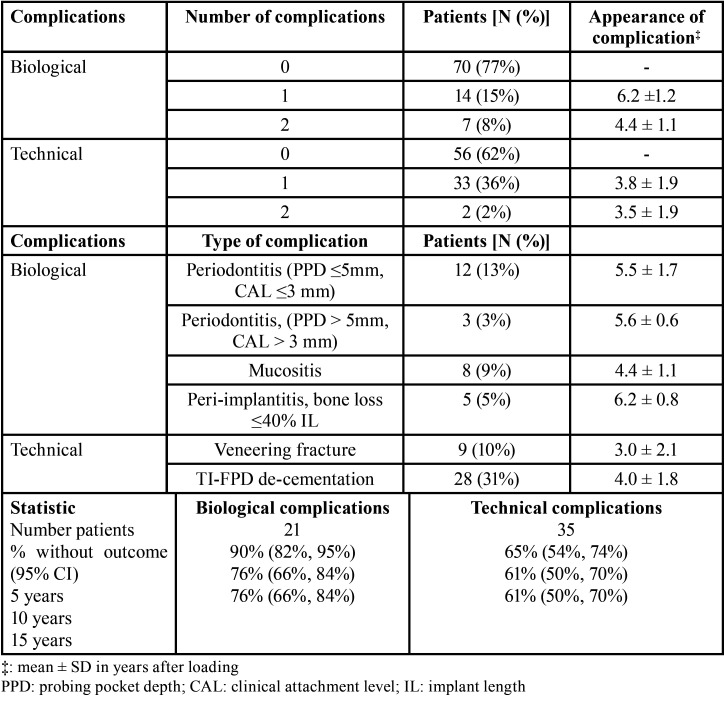
Appeared complications and survival outcomes.

**Figure 1:**
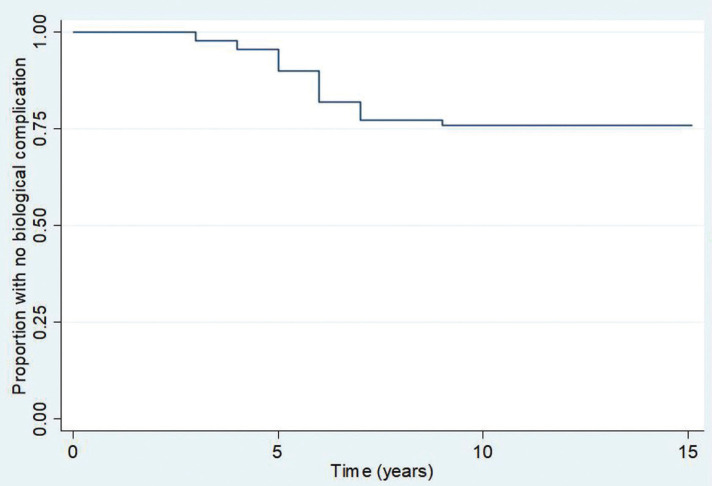
Kaplan-Meier graphic of biological complications over time.

**Figure 2:**
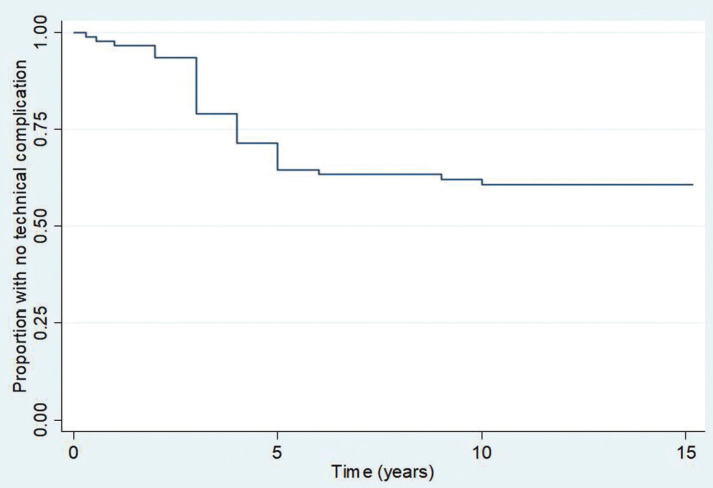
Kaplan-Meier graphic of technical complications over time.

**Figure 3:**
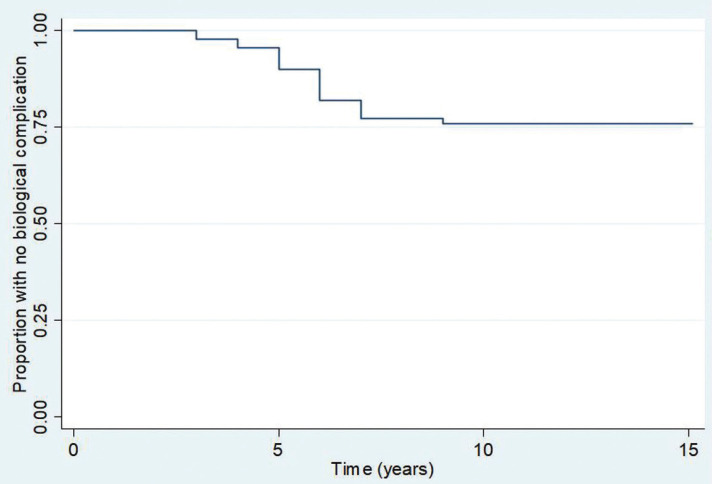
Kaplan-Meier graphic of biological complications over time by type of implant.

**Figure 4:**
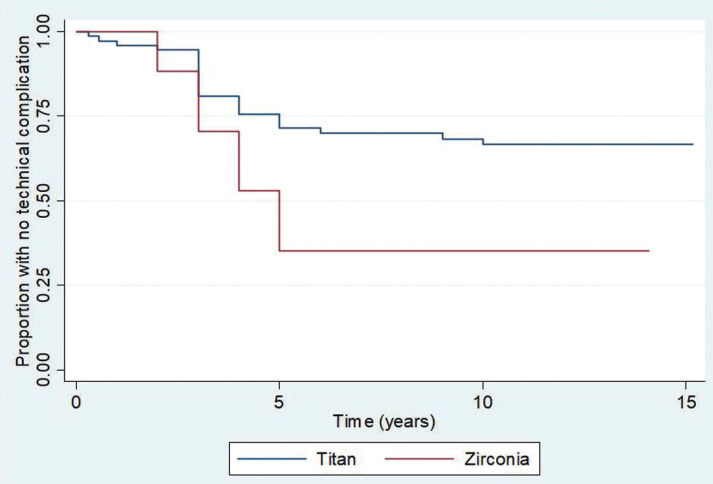
Kaplan-Meier graphic of technical complications over time by material used for the fabrication of the framework.

Regarding outcomes, survival analyses demonstrated that 19/21 (90%, 95%CI 82–95%), 16/21 (66%, 95%CI 66–84%), and 16/21 (66%, 95%CI 66–84%) patients were free of biological complications at 5 years, 10 years, and 15 years post-loading, respectively. Meanwhile, 23/35 (90%, 95%CI 54–74%), 21/35 (61%, 95%CI 50–70%), and 21/35 (61%, 95%CI 50–70%) patients were free of technical complications 5 years, 10 years, and 15 years post restoration, respectively (Table 4). The Kaplan-Meier graphs associated with these results are shown in Figures 1-4.

Analyses of potential effects of implant characteristics on the timing of biological complications and technical complications is shown in Table 3. The material used, Tit or ZI, had a significant effect on timing of technical complications, but only non-significant trends toward effects on the timing of biological complications. Restoration site represented by patient group (ANT, IpmTm, or ImTpm) was not significantly related to the timing of biological complications or technical complications. The implant system used (Straumann or Camlog) had a significant effect on the timing of biological complications, but no effect on the timing of technical complications. Significantly less restoration survival times without biological complications were observed with Straumann implants as opposed to Camlog implants. There was a trend toward lesser restoration survival time without biological complications for Tit frameworks than for restorations made of ZI. ZI frameworks also tended to yield lesser restoration survival times without technical complication. However, these comparisons were on the edge of significant consideration perhaps due to the limited number of patients (Table 3). Furthermore, no increased tooth mobility was observed and no association between the number of TI-FPD pontics and the appearance of biological or technical complications was observed at the time of TI-FPD removal.

## Discussion

The tooth-implant connection provides a biomechanical challenge. TI-FDPs have been shown to be a successful therapeutic modality (7,13). Previous studies comparing implant–implant retained and TI-FDPs have demonstrated comparable results regarding the technical and biological complications between these 2 treatment modalities (14,15). Several studies have reported failures and complications for TI-FPDs including abutment tooth and/or implant fracture, abutment screw loosening, tooth intrusion, caries, denture fracture or tooth devitalization (2-4,6,9,16,17). Such complications were also observed in referred cases in the author´s private clinic before patients were treated with the TI-FPD design presented in this cohort study. The data analysis of the 91 cases in this present study did not reveal abutment (tooth or implant) loss, fracture of TI-FPDs or abutments, abutment screw loosening or tooth intrusion. Furthermore, the results demonstrated that the time of appearance of biological or technical complications was not related to position of the implants or natural teeth abutments. The main reasons for treatment with TI-FPDs were anatomical (limited bone volume) and a more viable financial solution due to the reduced number of implants placed and bypassing augmentative surgical procedures at the patient’s request. For these reasons, the decision was to select a prosthetic restoration using a single implant combined with one natural tooth.

The use of rigid and non-rigid connections has been investigated in systematic reviews in which is underlined that the decision as to which connection should be chosen is often a matter of clinician preference and patient personal finances rather than scientific evidence (1,3,4,6).

In the present study, the results represent a survival rate of 100% for both implants and teeth. Other studies have reported an implant failure of 1.89% and 1.97% (after 5 and 10 years respectively) (3-6,15,18). Nickening *et al*. (17) in a 10 year-study found that 10% of teeth abutments had a biological complication and 23/449 of them were lost. Furthermore, they found that less than 5% of the implant abutments had a biological or a technical complication. Lang *et al*. (3) in their systematic review reported a TI-FPD survival rate of 94.1% after 5 years and 77.8% after 10 years of function and a cumulative failure rate of 5.9% after 5 years and 22.2% after 10 years. However, the majority of longitudinal implant studies have not distinguished between different types of restorations. Indeed, Brägger *et al*. (15) could not determine any evidence of a higher risk for technical or biological complications for TI-FPDs when comparing with FPDs retained only on implants or teeth.

It has been suspected that tooth intrusion depends on the level of periodontal support of the abutment teeth connected to implants by FPDs. Cordaro *et al*. (19) demonstrated that all cases with tooth intrusion occurred in patients having abutment teeth with normal periodontal support. Conversely, they found no tooth intrusion in patients with reduced periodontal support. Lindh *et al*. (13) reported de-cementation of 2 tooth abutments without any tooth intrusion. In recent reviews, Tsaousoglou *et al*. (1,6) reported no natural tooth intrusion in rigidly connected TI- FPDs and Ting *et al*. (1) concluded that intrusion was associated with lost or broken attachment or with loosening or fracture of the rigid connection (6,7,14,19).

Covering a natural tooth abutment with an AGC could provide improved protection of the abutment against caries and subsequent devitalization, and in case of de-cementation the AGC covering of the natural tooth acts as a “primary crown”. De-cementation and loss of TI-FPD retention was considered a technical complication if they appeared before or after the scheduled TI-FPD removal and was observed in 28 cases (31%). Patients did not notice this complication because the TI-FPDs maintained their retention on the abutments and were discovered at the next scheduled recall appointment. Several studies mention de-cementation as a common technical complication for a TI-FPD reporting a wide range from 9% Nickening *et al*. (17) to 37.3% Pjetursson *et al*. (18). According to the clinical protocol applied in this study, the provisionally cemented TI-FPDs were removed and subsequently re-cemented every 2 years for better review of the abutments and cleaning of the supra-structure. This practice was thought to provide improved security for the prevention of biological complications as clinical studies have identified success with regular supportive periodontal therapy in tooth-implant cases (20,21).

Bruxism as well as clenching teeth were exclusion criteria for patient selection in our study. However, in the present study 9 cases (10%) of veneering fracture were observed and bruxism and tooth clenching were exclusion criteria in the patients´ selection. The removal of the TI-FPDs every 2 years could be the reason for this complication in the present study population.

Patient factors related to oral hygiene, maintenance compliance, bruxism, and cleansability and caries prevention of the prosthetic design were considered in the treatment planning of the present study. Good oral hygiene is crucial for the longevity of both implants and teeth (1,3,15,16). All patients of the present study had 4 quarterly SPC follow-up appointments per year and had good oral hygiene (PL 15%, BOP 9%). However, 15 patients (16%) developed recurrent areas of active periodontitis (PPD > 5mm) that required further treatment, 5 (5%) were diagnosed with peri-implantitis and 8 (9%) developed peri-mucositis. Furthermore, 13% of abutment teeth at 5.5 years had PPD ≤5 mm and 3% of the teeth had PPD >5 mm at 5.6 years as well as increased probing depths in 5% of the implants 6.2 years after loading. These findings are in agreement with those of Gunne *et al*. (14) who reported bone loss of 0.2 to 0.4 mm between the 5th and 10th year. Interestingly, 6 patients had both a natural abutment with periodontitis (probing depth ≤5 mm) and an implant with mucositis and another patient had an implant with peri-implantitis (probing depth ≤5 mm) and a natural tooth abutment with periodontitis with a probing depth >5 mm. During subsequent treatment, provisional cement or calculus could not be detected. Brägger *et al*. (15) reported that 9.6% of TI-FPDs developed periimplantitis after a 5-year observation period. However, the Brager study does not consider whether an effective periodontal and implant maintenance protocol was implemented after delivery of the TI-FPDs. Lesser restoration survival times without biological complications were observed for Straumann implants than for Camlog ones. This could be the result of the higher number of Straumann (69) than Camlog implants (22) placed and the placement of Straumann implants in the posterior areas which may lead to enhanced cleaning difficulties for study patients.

Study limitations included a limited patient number, which resulted in some group comparisons with only a tendency rather than clinical significance; no full recording of the type/quality of opposing dentition and the absence of regular radiological examination due to country regulations on radiological assessment advising this could only be undertaken if clinical findings justified radiographic exposure.

The present study is from a specialist private practice-based retrospective analysis of existing clinical data. This could be helpful in identifying feasibility issues and designing a future randomized prospective study but may be effective when it evaluates efficacy of an intervention. All procedures performed in the present study were performed by the same clinician, a factor that may diminish the validity of the results.

Despite limitations of this study, the findings demonstrated that use of a contemporary designed TI-FPD could be used for tooth-implant connection in cases of partial edentulism to reduce high failure rates reported in other clinical studies (22). It therefore has the advantage of widening treatment modality options. When the clinician is confronted with treatment planning difficulties involving either patient financial restrictions or edentulous areas not conducive for placement of an adequate number of supporting implants including anatomical limitations such as inadequate alveolar bone for implant placement as well as risk assessment of the residual dentition, the splinting of dental implants to natural teeth may feasibly provide an option.
